# Prior knowledge based deep learning auto-segmentation in magnetic resonance imaging-guided radiotherapy of prostate cancer

**DOI:** 10.1016/j.phro.2023.100498

**Published:** 2023-10-10

**Authors:** Maria Kawula, Marica Vagni, Davide Cusumano, Luca Boldrini, Lorenzo Placidi, Stefanie Corradini, Claus Belka, Guillaume Landry, Christopher Kurz

**Affiliations:** a Department of Radiation Oncology, LMU University Hospital, LMU Munich, Munich, Germany; b Fondazione Policlinico Universitario “Agostino Gemelli” IRCCS, Rome, Italy; c Mater Olbia Hospital, Olbia (SS), Italy; d German Cancer Consortium (DKTK), Partner Site Munich, A Partnership Between DKFZ and LMU University Hospital Munich, Germany; e Bavarian Cancer Research Center (BZKF), Munich, Germany

**Keywords:** Auto-segmentation, Patient-specific models, Spatial transformer layer, Deep learning, MRgRT, MR-Linac, Prostate cancer

## Abstract

**Background and purpose:**

Automation is desirable for organ segmentation in radiotherapy. This study compared deep learning methods for auto-segmentation of organs-at-risk (OARs) and clinical target volume (CTV) in prostate cancer patients undergoing fractionated magnetic resonance (MR)-guided adaptive radiation therapy. Models predicting dense displacement fields (DDFMs) between planning and fraction images were compared to patient-specific (PSM) and baseline (BM) segmentation models.

**Materials and methods:**

A dataset of 92 patients with planning and fraction MR images (MRIs) from two institutions were used. DDFMs were trained to predict dense displacement fields (DDFs) between the planning and fraction images, which were subsequently used to propagate the planning contours of the bladder, rectum, and CTV to the daily MRI. The training was performed either with true planning-fraction image pairs or with planning images and their counterparts deformed by known DDFs. The BMs were trained on 53 planning images, while to generate PSMs, the BMs were fine-tuned using the planning image of a given single patient. The evaluation included Dice similarity coefficient (DSC), the average (HD_avg_) and the 95th percentile (HD_95_) Hausdorff distance (HD).

**Results:**

The DDFMs with DSCs for bladder/rectum of 0.76/0.76 performed worse than PSMs (0.91/0.90) and BMs (0.89/0.88). The same trend was observed for HDs. For CTV, DDFM and PSM performed similarly yielding DSCs of 0.87 and 0.84, respectively.

**Conclusions:**

DDFMs were found suitable for CTV delineation after rigid alignment. However, for OARs they were outperformed by PSMs, as they predicted only limited deformations even in the presence of substantial anatomical changes.

## Highlights

• First study to investigate deep learning models predicting vector fields for organs-at-risk segmentation in magnetic resonance imaging-guided radiotherapy for prostate cancer.
• Personalized models improve organ-at-risk segmentation compared to generic baseline models.
• Deformation networks are not suitable for organs undergoing substantial volume changes.

## 1 Introduction

Integrated magnetic resonance (MR) linear accelerators (MR-Linacs) facilitate daily MR image (MRI) acquisition and dose adaptation [1], [2], [3]. This enables the reduction of safety margins and hypofractionation [4], [5], [6], [7]. However, online adaptive MR-guided radiation therapy (MRgRT) has longer workflows than conventional linacs. The most time-consuming step besides irradiation is the generation of updated organ-at-risk (OAR) and target volume contours on the fraction MRIs [8], [9], [10].

In clinical workflows [1], the planning and fraction MRIs are first rigidly aligned and then registered with deformable image registration (DIR) in the treatment planning system (TPS). The clinical target volume (CTV) is rigidly copied to the daily MRI, while the OARs are propagated with a displacement vector field estimated in the TPS. Since the quality of propagated contours is suboptimal for dose evaluation and optimization, manual corrections are required. Automatic segmentation could shorten the adaptation time as suggested by Cha et al. [11] or Zabel et al. [12], and reduce inter- and intra-physician variability [13], [14].

Despite several studies on MRI segmentation [15], [16], the utilization of planning contours to enhance auto-segmentation on fraction images is still limited. Fransson et al. [17] showed single-patient 2D models trained with the first fraction image to predict contours on consecutive treatment days. Li et al. [18] used a similar 2D training strategy, but suggested refining the model after each irradiation. Kawula et al. [19] proposed using the planning image to fine-tune generic models, which adjusted the model to the patient of interest without losing generality. Eppenhof et al. [20] presented another strategy for CTV segmentation based on the prediction of a dense displacement field (DDF) between the planning and fraction images. Our work is the first study investigating this method for the segmentation of OARs in prostate cancer patients.

This study thus aimed to train models predicting DDFs (DDFMs) between the planning and fraction images, enabling propagation of planning contours to the daily anatomy. Moreover, patient-specific models (PSMs) [19] were trained by fine-tuning generic models (baseline models, BMs) using segmented planning MRIs. All methods were compared to DIR with contour propagation and rigid contour copying.

## 2 Materials and Methods

### 2.1 Dataset

Datasets from two facilities were used: 73 patients from the Gemelli University Hospital in Rome formed the first cohort (C1). For ten patients the planning and one fraction MRI were available, while for the remaining 63, only the planning image was included. The second cohort (C2) had 19 patients from the LMU University Hospital. Patients in C2 had one planning and 5–33 fraction images (240 fraction MRIs in total). Informed written consent was obtained from all patients and the study was carried out carried out in accordance with relevant ethics guidelines and regulations (LMU: ethics project number 20–291, Gemelli: EC authorization number 3460).

All delineations used as ground truth were created during the clinical workflow. Rectum, bladder, and CTV contours on planning MRIs (planning contours) were manually drawn by physicians. At every fraction a new MRI was acquired for potential plan adaptation. A radiation therapy technologist (RTT) performed manual rigid registration of the images, prioritizing the alignment of the prostate and seminal vesicles, followed by the adjustment of bones and outline. Subsequently the planning and fraction images were registered with DIR in the TPS. The resulting DDF was used to propagate the planning OAR contours to the daily MRI. Usually, a physician manually corrected them due to their insufficient quality, focusing mostly on the high-dose region, i.e. the proximity of the planning target volume (PTV). The resulting delineations will be referred to as fraction contours. In our previous work [19], no differences in OAR annotation styles were found between the two cohorts. The C2 CTV contours included slightly more normal tissue than the C1 CTV structures, however, differences were subtle. The percentages of low, intermediate and high-risk cases, which determine the CTV contour size, were alike in both cohorts.

All MRIs were acquired at 0.35 T MR-Linacs (MRIdian, ViewRay Inc, Cleveland, Ohio) with a balanced steady-state free precession (bSSFP) sequence resulting in T2∗/T1 contrast [1]. The in-plane spacing was 1.5 mm × 1.5 mm and the axial slice thickness was 1.5 mm (∼90% of the MRIs in both cohorts) or 3 mm (remaining ∼10%). The latter were resampled to 1.5 mm slice thickness using the open-source tool Plastimatch [21] with either linear (for images) or nearest neighbor (for contours) interpolation. To mimic the manual rigid alignment done clinically by RTTs, all images were cropped to 192×192×192 voxels around the CTV centroid.

### 2.2 Architectures

For each anatomical structure, a separate network was trained. The MONAI [22] implementation of a 3D U-Net architecture [23] was used for segmentation with all investigated models. It comprised five resolution levels, i.e., there were four down- and four up-sampling operations. Each level had two convolutions with 3×3×3 kernels, followed by instance normalization and PReLU activation. The down- and up-sampling employed double-stride and up-convolution, respectively.

For BMs and PSMs, the U-Net was predicting which voxels of the input image belong to the foreground. For DDFMs it was predicting the DDF of size 192×192×192×3 between the planning and the fraction image. The output of the DDFM U-Net was given to a spatial transformer layer (STL) [24] that served as a grid interpolator and had no learnable parameters.

### 2.3 Training of DDFMs

**Fig. 1:**
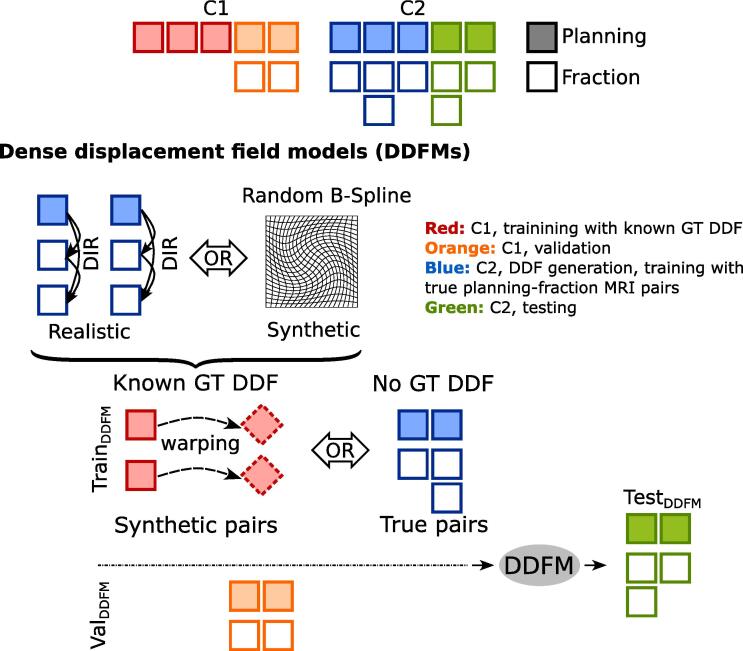
DDFM training and evaluation scheme. Cohorts 1 (C1) and 2 (C2) were utilized in the study. In the first approach of the DDFM training with known ground truth (GT) DDFs, planning-fraction image pairs were generated by warping the C1 MRIs using either the “realistic” or the “synthetic” dense displacement fields (DDFs). The former were derived from deformable image registration (DIR) of the C2 images. The second approach had no ground truth (GT) DDF but was trained on the true planning-fraction image pairs from C2. Both variants were validated and tested on the remaining patients from C1 and C2, respectively.

Two types of input data were considered. The first type were the C1 planning images and contours warped by known training DDFs generating synthetic fraction images and contours. The second type were the true planning-fraction image pairs of C2 patients (125 pairs for training and 115 pairs for testing), without a ground truth DDF. In both scenarios ten C1 patients, for whom fraction data were available, were used as an independent validation set. Figure 1 illustrates the data split and the DDFM training scheme.

Training DDFs were generated in two ways. For the *synthetic* DDFs, TorchIO’s [25] RandomElasticDeformation function was employed on-the-fly during training, separately for each mini-batch. Its parameters were set to yield clearly visible transformations (number_of_control_points = 10, max_displacement = 35 mm) due to the expected substantial volume changes in the OARs. The so-called *realistic* DDFs were extracted from the DIR between all image pairs within a patient’s dataset (planning and fraction MRIs). This was done for ten C2 patients using Plastimatch with multistage B-spline registration [26] and resulted in a total of 860 different realistic DDFs. Information on the B-spline deformation are in the supplementary material (section A).

DDFM training was carried out in two ways. The first followed the progressive training scheme described by Eppenhof et al. [27]. The network was trained at different resolutions, starting with a coarse alignment of *n -1* = 4 times sub-sampled MRIs (corresponding to *n* = 5 U-Net levels) and gradually adding higher-resolution data as the training progressed. In the second variant, the network was trained at once.

During training, either true or the synthetic input data pairs were used without data augmentation. For the approach with the synthetic image pairs, either the synthetic or the realistic DDFs were used for model training. The training variants are shown in Figure 1.

The total loss function $L_{\mathrm{tot}}$ was defined as follows:

(1) $$L_{\mathrm{tot}}=\lambda_{\mathrm{DDF}}L_{\mathrm{DDF}}+\lambda_{\mathrm{seg}}L_{\mathrm{seg}}+\lambda_{\mathrm{img}}L_{\mathrm{img}}\text{.}$$

**Fig. 2:**
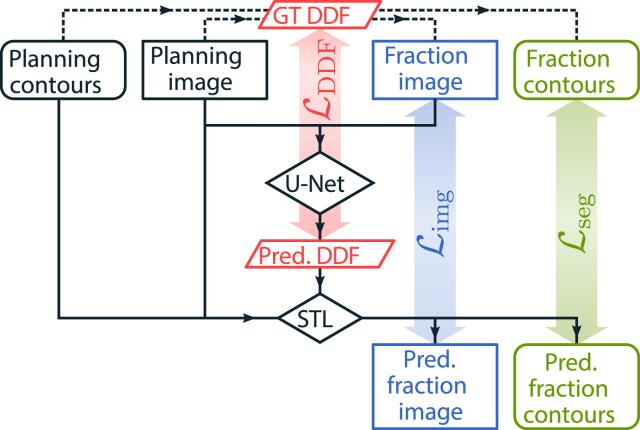
Training scheme and possible loss terms L. The dense displacement field (DDF) predicted by the U-Net is passed to the spatial transformer layer (STL) along with the planning data to generate predictions for the fraction image and contours. The usage of the data variant with the synthetic planning-fraction pair and the known ground truth (GT) DDF has been indicated with dashed lines.

The DDF term $L_{\mathrm{DDF}}=\lambda_{\mathrm{DDF},L2}L_{2}+\lambda_{\mathrm{DDF},\mathrm{reg}}L_{\mathrm{reg}}$ measured the similarity between the ground truth and the predicted DDF using the $L_{2}$ norm and regularized the predicted DDF with the bending energy $L_{\mathrm{reg}}$ [28]. For the true planning-fraction image pairs, there was no ground truth DDF, and therefore no $L_{2}$ term. The segmentation loss $L_{\mathrm{seg}}$ quantified the similarity between the contours with the (multi-scale) Dice Similarity Coefficient (DSC) [29]. The DSC-based loss function was chosen due to the class imbalance with relatively few voxels belonging to the foreground (OARs and CTV), and the majority belonging to the background. The image loss $L_{\mathrm{img}}$ aimed to optimize the similarity between the registered images using either cross-correlation or $L_{2}$ norm. The λ coefficients determined the contribution of each term in the total loss. All training variations are depicted in Figures 1 and 2. Among the investigated DDFMs the best DSC for the ten C1 validation images was obtained while training the entire network at once (no progressive learning) with true planning-fraction image pairs, and the loss function including terms with bending energy loss, multi-scale DSC, and $L_{2}$ norm for image similarity. The optimal weighting parameters were found to be $\lambda_{\mathrm{DDF}}=1,\lambda_{\mathrm{DDF},L2}=0,\lambda_{\mathrm{DDF},\mathrm{reg}}=10,\lambda_{\mathrm{seg}}=100,\lambda_{\mathrm{img}}=1$, and the number of epochs to be 200 epochs. The training was carried out on Nvidia Quadro RTX 8000 or Nvidia RTX A6000 GPUs (used also later for the BM and PSM training) and took two days. No signs of overfitting were present at that stage, and the DDFM predictions did not improve significantly beyond that point. All results presented later were obtained using the settings listed above.

### 2.4 Training of baseline and patient-specific models

**Fig. 3:**
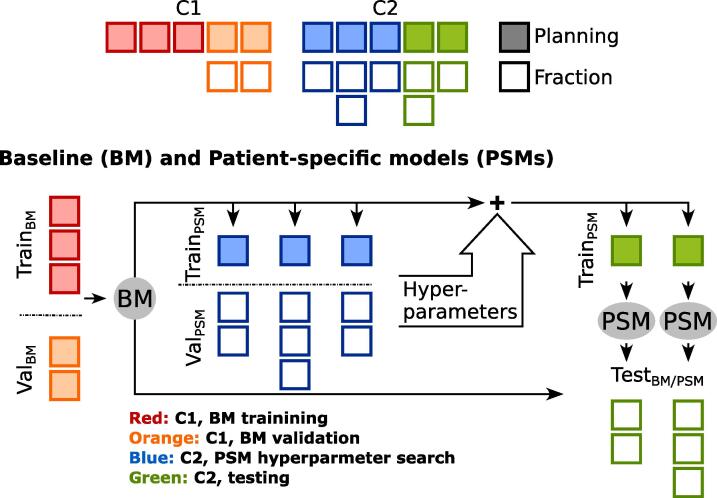
Baseline (BM) and patient-specific model (PSM) training and evaluation scheme. Cohorts 1 (C1) and 2 (C2) utilized in the study. The BM was trained and validated on MRIs from C1. For the PSMs, a hyperparameter search was conducted using MRIs from ten C2 patients. The final PSMs were generated through fine-tuning the BM with planning MRIs of the 9 C2 test patients with fixed hyperparameters. Both methods shared the same test set of fraction data of these 9 C2 patients.

For the single-label BMs, three 3D U-Net models have been trained on 53 planning images from 53 C1 patients for bladder, rectum, and CTV segmentation. BM networks were trained over 300 epochs with a batch size of two for 20 h. Patient-specific transfer learning applied to the BMs. For each patient, the BM was fine-tuned with its on-the-fly augmented planning image and contours. Hyperparameters were adjusted using ten C2 patients. Figure 3 shows the training scheme. PSMs did not benefit from fine-tuning beyond 500 epochs which took 3 h. There were small improvements in DSC after 300 epochs, where training could have stopped. For the remaining nine C2 cases (the same as for the DDFMs) PSMs were fine-tuned over 500 epochs with fixed hyperparameters for the final testing. The process of hyperparameter optimization was described in detail by Kawula et al. [19].

### 2.5 Benchmarking and data evaluation

For benchmarking, a classical multistage B-spline registration with Plastimatch and rigid copying of the planning contours to the daily anatomy were performed (see supplementary material section A).

All investigated methods shared the same test set of nine C2 patients having 115 fractions in total. Network predictions were compared to the ground truth via DSC, the 95^th^ percentile (${\mathrm{HD}}_{95}$) and the average (${\mathrm{HD}}_{\mathrm{avg}}$) Hausdorff distance (HD), calculated using Plastimatch. To prevent bias towards patients with more fractions, we initially calculated the average DSC and HDs for each patient. The final model performances were then reported as the averages over all test patients. Since the calculated metrics (average values of DSC and HDs for each patient) followed a normal distribution, as determined by the Kolmogorov–Smirnov test, we computed the mean values along with their corresponding standard deviations. Evaluation of rectum segmentations considered slices including the PTV and ten additional slices reaching 1.5 cm above and below the upper and lower PTV ends. To assess statistical significance of differences among the methods the paired t-test was conducted for all three metrics, employing a significance level of 0.05.

## 3 Results

**Fig. 4:**
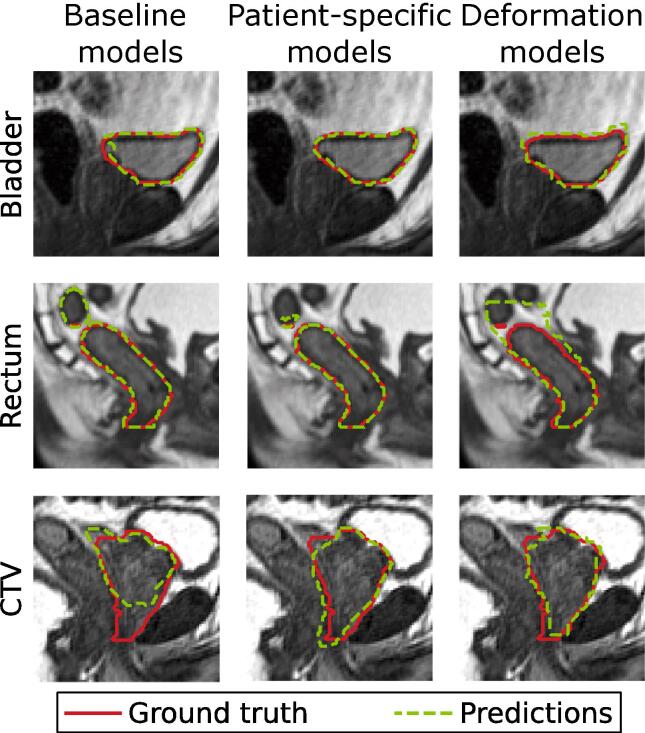
Sagittal view of exemplary test patients showing the comparison between segmentations performed by (left) the baseline models, (middle) patient-specific models, and (right) dense displacement field (deformation) models for (top) the bladder, (middle) rectum, and (bottom) clinical target volume (CTV). Rectum contours are shown only within the evaluation volume, i.e. slices including the planning target volume (PTV) and 1.5 cm above and below its upper and lower ends.

Fig. 4 shows sagittal slices from exemplary test patients with the predictions of BMs, PSMs, and DDFMs versus ground truth segmentation. All methods segmented the bladder similarly well. For the rectum the DDFM did not capture its volume increase with respect to the planning day, while the BM did not determine the cranial end correctly. PSM and DDFM delineated the CTV well, but the BM had larger deviations from the ground truth.

The average DSC, ${\mathrm{HD}}_{95}$, and ${\mathrm{HD}}_{\mathrm{avg}}$ from comparing the segmentations generated by the investigated methods and the ground truth delineations are given in Table 1. For the OARs, the PSMs gave the highest mean DSC of 0.91/0.90 for the bladder/rectum. BMs yielded slightly lower mean DSC values of 0.89/0.88 for bladder/rectum, however the difference for the rectum was not statistically significant. Among the three examined deep learning approaches, the DDFMs delivered the lowest (in all cases statistically significant) DSC with a mean of 0.76 for both OARs. ${\mathrm{HD}}_{\mathrm{avg}}$ and ${\mathrm{HD}}_{95}$ showed a similar trend as the DSC assessment, however, not all differences were statistically significant. Conventional DIR with Plastimatch slightly outperformed the DDFM for the OARs, however the differences were not statistically significant. The results of the statistical analysis are provided in the supplementary material (section B).

**Table 1:** Mean and (standard deviation) of Dice similarty coefficient (DSC), 95^th^ percentile (${\mathrm{HD}}_{95}$), and average (${\mathrm{HD}}_{\mathrm{avg}}$) Hausdorff distance for the test set patients. The dense displacement field (DDFM), and patient-specific models (PSM) [19] are compared to the baseline models (BMs), conventional deformable image registration with Plastimatch, and rigid copying of the planning contours to the fraction anatomy. The analysis was performed for the nine C2 test patients (in total 115 fractions). The results of the best-performing models are in bold.

**Method**	**Bladder**	**Rectum**	**CTV**
	DSC	DSC	DSC
	${\mathrm{HD}}_{95}$ [mm]	${\mathrm{HD}}_{95}$ [mm]	${\mathrm{HD}}_{95}$ [mm]
	${\mathrm{HD}}_{\mathrm{avg}}$ [mm]	${\mathrm{HD}}_{\mathrm{avg}}$ [mm]	${\mathrm{HD}}_{\mathrm{avg}}$ [mm]
Plastimatch	0.79(0.14)	0.78(0.08)	0.83(0.11)
	9.6(4.7)	7.4(4.1)	4.5(2.3)
	3.6(2.0)	2.3(1.0)	2.3(2.2)
Copying	0.72(0.11)	0.70(0.03)	**0.89(0.02)**
	12(4)	8.8(3.2)	2.9(0.9)
	4.8(1.6)	3.1(0.8)	1.1(0.4)
BM	0.89(0.07)	0.88(0.03)	0.62(0.24)
	5.9(4.2)	4.8(1.7)	11(4)
	1.8(0.9)	1.4(0.4)	4.1(1.9)
DDFM	0.76(0.09)	0.76(0.03)	0.87(0.06)
	11(3)	5.0(1.7)	**2.3(0.7)**
	4.1(1.3)	1.7(0.3)	**1.0(0.5)**
PSM	**0.91(0.07)**	**0.90(0.02)**	0.84(0.07)
	**4.0(2.6)**	**3.6(0.8)**	4.0(0.8)
	**1.4(0.7)**	**1.1(0.2)**	1.6(0.3)

Apart from the BM, all methods gave similar results for the CTV. The highest DSC was observed for the rigidly copied contours, while the best HDs were achieved for DDFMs. Nevertheless, for the DSC and ${\mathrm{HD}}_{\mathrm{avg}}$ the differences between Plastimatch, copying and DDFMs lacked statistical significance.

**Fig. 5:**
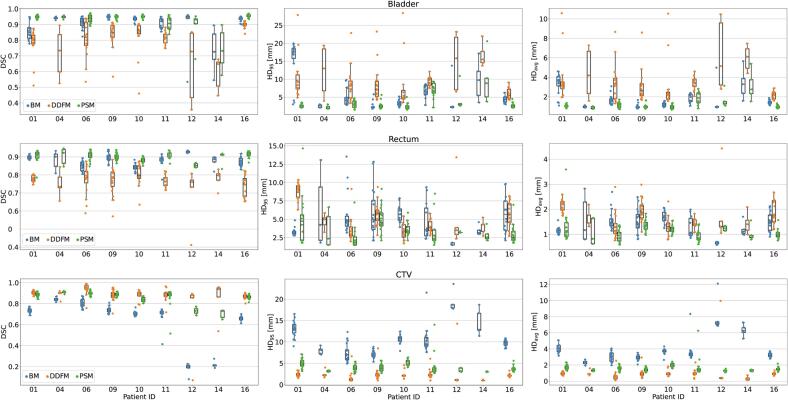
Results for (top) bladder, (middle) rectum, and (bottom) clinical target volume (CTV) comparing the predictions of (blue) the baseline, (orange) deformation vector field, and (green) the patient-specific training for the nine test patients. A single point on the plot represents (left) the Dice similarity coefficient (DSC), (middle) 95^th^ percentile Hausdorff distance (${\mathrm{HD}}_{95}$), and (right) average Hausdorff distance (${\mathrm{HD}}_{\mathrm{avg}}$) for a predicted fraction contour.

Fig. 5 illustrates the DSC and HDs separately for each test patient. In most cases, the PSMs worked best and improved the BMs predictions considerably. The exceptions were patients 12 and 14, showing considerable differences in bladder filling between the planning and fraction days. For the bladder, the DDFMs performed notably worst, but in terms of HDs for the rectum they outperformed BMs in four out of nine cases.

## 4 Discussion

For OARs, the PSMs gave the best outcomes in terms of DSC and HDs, comparable to the state-of-the art in automatic pelvic segmentation [17], [30], [31], [32]. For the bladder, PSMs mostly corrected larger volume misclassifications of the BM as observed in patient 01. For the rectum, the PSMs accurately identified the superior and inferior ends on fraction MRIs, in agreement with the planning contours. However, this intrinsic feature might hinder PSM performance for organs that are likely to change shape in the course of treatment or in the case of imprecise planning contours. This is evident in Fig. 5, patient 12, who did not follow the drinking protocol consistently. For OARs, the DDFMs did not perform satisfactorily. Regardless of the presence or absence of the regularization term during training the predicted fields were limited to small deformations. Moderate volume differences in OAR fillings, which could easily be captured by the PSMs, were not predicted by the DDFMs. The presence of substantial anatomical changes requires the DDFMs to model long-distance relationships between voxels, while in segmentation tasks (BMs, PSMs), local information is more critical. This is in agreement with the work of Luo et al. [33] which suggests that due to the limited effective receptive field of convolution operations their ability to model long-range spatial relations is limited. Moreover, it has been demonstrated in Li et al. [34] that as the convolutional layers deepen, the impact of far-away voxels lowers quickly. This problem could be potentially solved by architectures based on transformers, as the intrinsic self-attention mechanisms have larger effective receptive fields, making them capable of capturing long-range spatial information [35].

Nevertheless, the potential benefit of DDFMs over the BMs lies in the utilization of the planning delineations as the starting point for the predicted contours. Aside from the rectum, this may be beneficial for other tubular organs, e.g. the esophagus or spinal canal, where physicians choose to contour only a section of the organ near the PTV. This benefit can be seen in Fig. 5 where the rectum HDs for several patients is lower for DDFM than for BM.

The advantage of using planning segmentation as a starting point for the fraction contours, applies also to the conventional DIR by Plastimatch. Both methods yielded similar DSC and HDs. However, the DIR took approximately 1 min to register a pair of images and deform one contour set, while the generation of DDFM contours required 1-2 s.

For the CTV segmentation, the methods involving planning contours in some way (all but BM) performed similarly well, with rigid copying having the highest DSC and DDFMs showing the best HDs. However, cropping of all images around the CTV centroid most likely led to a better performance of contour copying than could have been achieved clinically. The high performance of methods utilizing planning contours was to be expected due to the way prostate CTV is delineated in clinical practice. To avoid unexpected changes from the applied deformations, the planning CTV contours are rigidly copied to the fraction anatomy and only slightly adjusted, if necessary. Similar good performance of DDFMs for CTV has been confirmed by Eppenhof et al. [20], showing DSC/${\mathrm{HD}}_{95}$ of 0.86/5.66 mm. However, we are not aware of any studies showing high performance of DDFMs for OARs.

There are some limitations of this study. Only the two key OARs for prostate cancer patients were considered. Nevertheless, the methods should be applicable to other organs. Moreover, bladder and rectum undergo considerable volumetric changes, and serve as an excellent evaluation scenario for the networks considered in this work. The dataset size is another potential limitation. For most patients only the planning MRI was included due to the tedious process of data export and to the best of our knowledge, the are no publicly available datasets collecting MR-Linac data. Additionally, only geometric assessment with DSC and HDs has been provided, while a qualitative analysis such as physician’s grading [19] could better gauge the clinical value of the predicted contours. Finally, some OAR ground truth fraction contours showed sub-optimal quality, as time constraints led to corrections being applied primarily around the PTV. Based on the geometric metrics and our visual inspection, none of the investigated segmentation methods appear fully reliable, necessitating physician’s inspection before clinical use. Nevertheless, prior studies suggest, that correcting deep learning contours is faster than manual contouring from scratch [11], [12].

To summarize, on average patient-specific U-Net models (PSMs) improved segmentation compared to BMs. DDFMs predicted only limited deformations and achieved good results for the CTV, while being less suitable for organs undergoing substantial volume changes. As a next step, transformer models [36] involving attention mechanisms will be investigated as an alternative to DDFMs. Another method to be explored will be U-Nets taking the planning MRI with manual contours as an additional input [37] to aid segmentation on fraction images.

## Ethics statement

All LMU patients provided informed written consent within the scope of an ethics approved study protocol in place at the Department of Radiation Oncology of the LMU Munich University Hospital (ethics project number 20–291).

Images from Fondazione Policlinico Universitario “Agostino Gemelli” were collected under EC authorization number 3460 for image analysis.

## CRediT authorship contribution statement

**Maria Kawula:** Investigation, Software, Formal analysis, Data curation, Writing - original draft, Visualization. **Marica Vagni:** Data curation, Writing - review & editing. **Davide Cusumano:** Data curation, Writing - review & editing. **Luca Boldrini:** Data curation, Writing - review & editing. **Lorenzo Placidi:** Data curation, Writing - review & editing. **Stefanie Corradini:** Supervision, Writing - review & editing. **Claus Belka:** Supervision, Writing - review & editing. **Guillaume Landry:** Conceptualization, Writing - review & editing, Supervision. **Christopher Kurz:** Conceptualization, Writing - review & editing, Supervision, Funding acquisition.

## Declaration of Competing Interest

The authors declare that they have no known competing financial interests or personal relationships that could have appeared to influence the work reported in this paper. The Department of Radiation Oncology of the University Hospital of LMU Munich has a research agreement with ViewRay. ViewRay did not fund this study and was not involved and had no influence on the study design, the collection or analysis of data, or on the writing of the manuscript.
